# Investigating the Role of Remote Working on Employees’ Performance and Well-Being: An Evidence-Based Systematic Review

**DOI:** 10.3390/ijerph191912373

**Published:** 2022-09-28

**Authors:** Bruna Ferrara, Martina Pansini, Clara De Vincenzi, Ilaria Buonomo, Paula Benevene

**Affiliations:** Department of Human Studies, Libera Università Maria SS. Assunta, 00193 Rome, Italy

**Keywords:** employees wellbeing, work-health balance, occupational health promotion, innovative, intrapreneurial skills, positive attitudes at work, smart learning, working environments

## Abstract

Remote working refers to a working model in which employees can pursue work tasks outside the organization due to the use of technology. Several research papers showed that different assumptions are linked to remote work because of the flexibility and autonomy granted to employees when working remotely or from home. This review consistently aims to describe remote work’s role in employees’ well-being and performance. Using the Preferred Reporting Items for Systematic reviews and Meta-analyses (PRISMA) guidelines, 20 peer-reviewed papers published from 2010 until 2021 were selected for this review. Findings showed various and mixed consequences on employees’ performance and well-being. Specifically, remote working affects employees’ perceptions about themselves and their workplaces and contributes to their physical and mental health, particularly regarding work-life balance. Managerial implications for remote working implementation will be discussed in the paper.

## 1. Introduction

According to Eurofound 2020 [1] estimates, approximately 40% of employees in the EU began to telework full-time due to the pandemic. In the EU, teleworkers were approximately 5% of all employees in 2000 [2], and before the outbreak, just 15% of the employed in the EU had ever worked from home [3].

While remote work (or work from home) was not introduced because of the pandemic, the healthcare emergency pushed enormously towards this shift. Therefore, adopting new forms of work organization based on flexibility and autonomy of the employees in terms of the places and times of work was a strategic need for organizations facing COVID-19 [4].

There is no universally accepted definition of telework [5]. Instead, according to the studies published in the last two years, different names covering different patterns of new ways of working emerged. In most cases, the same term is used for several types of work, leading to overlaps. Apart from telework, other terms referring to workers partly or entirely performing their tasks and duties outside of the office are: work from home [6,7,8], remote working [9,10,11,12,13], telecommuting [14,15,16], or e-working [17]. These constitute the most recurrent terminology covering different patterns of work organization outside the workplace. Despite the lexical issue, all these terms are built around two main concepts: (1) being physically outside the organization’s premises while working, and (2) being able to pursue the tasks due to technology. In this work, we will use the abovementioned terms interchangeably. Apart from the definition issue, the amount of weekly work schedule arranged as remote working depends on each organization and, eventually, the choice can be left to employees [18]. Such solutions have proven to decrease office costs (e.g., related to heating, office size, and premises) [19] and be linked to sustainability outcomes (e.g., lower traffic congestion and pollution) [20].

Several assumptions are linked to remote work because of the flexibility and autonomy granted to employees when working from home. First, it is assumed that remote work may lower employees’ stress and burnout, and reduce work fatigue and work-home conflict, thus enhancing employees’ work engagement and job satisfaction which, in turn, increases job performance [21].

On the other hand, there are other assumptions about the potential drawbacks of remote work at both individual and organizational levels. For example, employees working from home might face problems linked to poor ergonomic facilities, leading to muscular pain or even work accidents, or they might work longer than in the office due to the lack of a specific time frame for the end of work [22]. More than that, work isolation may jeopardize employees’ sense of belonging to their organization [23] and compromise receiving social support from colleagues, increasing the risk of stress and anxiety [22].

Overall, current research defining what remote working is and its theoretical implications provide mixed results regarding the potential effects of this arrangement on organizational processes and employees’ well-being and productivity.

At the same time, the last two years required organizations to massively use ICT-based solutions, which implied workplace-relevant changes in HR practices and organizational models, due to the higher flexibility required [24]. Significant challenges are posed by the need to set up new ways to monitor and assess performance and recognize individual and team results. Remote work cannot allow the direct monitoring and assessment of the work done during the time spent in the office [4]. At the same time, the unprecedented times in which this shift occurred did not allow organizations to clearly structure such processes [24]. Similarly, it is likely that the effects of telework on employees follow the same mixed patterns, with some employees feeling higher well-being in a flexible work environment [21], and others suffering from the reduced attendance in the office [23].

Remote work is due to stay after the emergence of the pandemic, and organizational changes are far from being concluded [25]. However, the speed and the rate of the shift toward partial or total remote work depends on several factors. These include the technological readiness of the organizations, the quality of services and technological tools put at the employees’ disposal, the employees’ skills and competencies in dealing with these new ways of working, and the feasibility of dislocating tasks and duties outside the office.

Undoubtedly, time, working conditions and organizational processes are linked to substantial differences in teleworkers’ performance and well-being. At the same time, it is essential to deepen the knowledge about which evidence is rising from the research about the risks and opportunities linked to teleworking and which conditions and settings shape the teleworkers’ satisfaction, well-being, safety, and performance [18].

To sum up, the spread of papers describing the role of teleworking on employees generated a massive amount of knowledge, that is potentially helpful for managers and researchers to individuate good practices to make this new work arrangement sustainable for individuals and organizations. At the same time, findings on the effects and the applicability of remote working are still mixed. For these reasons, this paper aims to provide a systematic literature review of the risks and opportunities for employees’ stress and well-being when teleworking, as they emerged in papers published from 2010 to 2021.

## 2. Methods

Following the PRISMA guidelines, we searched for English papers, peer-reviewed and published in a time range from 2010 until 2021. Regarding the exclusion criteria, publications different than research articles in peer-reviewed journals and conceptual papers on remote working were considered unrelated to this review.

### 2.1. Information Sources and Search Strategy

Databases and search engines, such as EBSCOhost, ProQuest, and Web of Science, were employed for the search, which took place from May 2021 to June 2022, and applying the following combination of keywords: “remote working” or “telework” or “eworking” or “e-working” or “work from home” or “home-based tele-work” or “virtual working” or telecommuting or “smart working” or “agile working” or “agile work” or “smart work” or “teleworking” or “ework” or “e-work” or “home working” or “home work” or “home-based work” or “home based work” or “home-based working” or “home based working” “home-working” or “home-work” AND “Psychosocial risks” or “well-being” or “well-being” or “stress” or “technostress” or “tecnostress”

The keywords covered two main topics: the definition of remote working and psychosocial dimensions. These were searched in the publication title or/and abstract or/and paper keywords.

### 2.2. Data Collection Process

**Figure 1:**
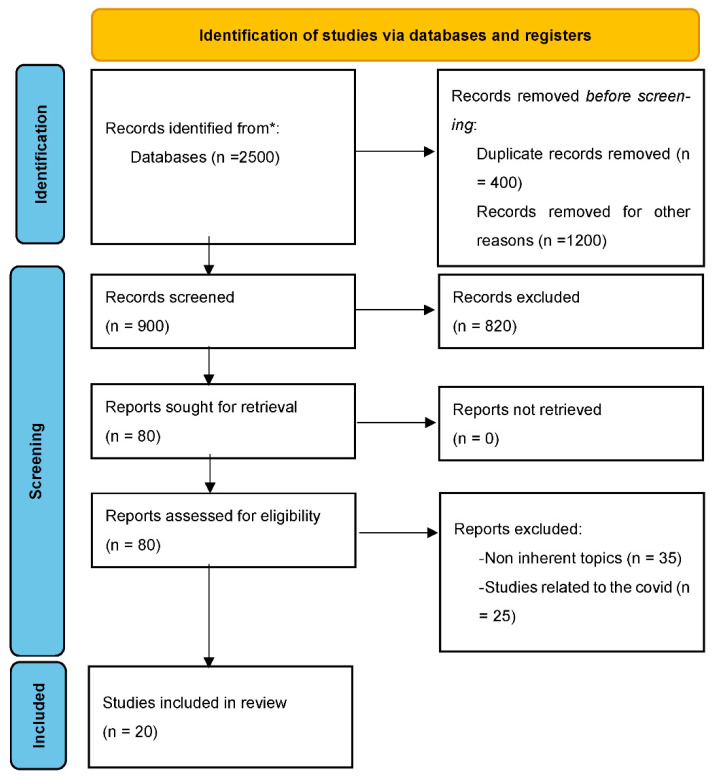
PRISMA 2020 flow diagram for new systematic reviews which included searches of databases and registers only. * Consider, if feasible to do so, reporting the number of records identified from each database or register searched (rather than the total number across all databases/registers).

As shown in Figure 1, after applying the inclusion and exclusion criteria, a final number of 20 papers was deemed suitable for this review. Table 1 reports for each paper information about the methods (study methodology, sample), the teleworking definition, and the tested relationships.

## 3. Results

The details reported in Table 1 confirm the high variability in terms of types of organizations, participants’ characteristics, and remote working definitions reported in the current literature. Nevertheless, it is still possible to individuate some patterns of influence on employees’ health and well-being.

### 3.1. Remote Working Influences the Perceptions of Oneself at Work and of the Workplace

Several studies showed that teleworkers experienced higher job satisfaction [32,34], work engagement, and motivation [45,46]. Such effects are direct or mediated by dynamics related to personal life (i.e., lower work-life conflict because of the higher time spent at home; [26]) or to organizational life (i.e., high support from colleagues during teleworking improves beliefs about one’s work conditions; [32,39] Other outcomes mentionable in this category regard organizational commitment [34], enthusiasm [34], and sense of comfort [45]: the higher the frequency of teleworking, the higher the likelihood for workers to experience such conditions.

Some studies reported positive effects of teleworking on perceived performance [40,45,46]. Since well-being and productivity are also connected, teleworking thus increases job and life satisfaction. Life satisfaction partially mediates the relationship between telework and productivity, so the higher the life satisfaction, the higher the productivity [47]. Among the selected studies, only Suh and colleagues [37] reported a negative effect, showing that the higher the intensity of telework, the higher the strain, and the lower the job satisfaction. Overall, being a teleworker is generally associated with positive outcomes related to the beliefs and emotions about oneself and one’s life. This is particularly true when teleworking reduces the strain from balancing personal and professional life and when the organization supports this arrangement formally and informally.

Compared to the effects on individual beliefs, findings regarding employees’ perceptions of the workplace are more heterogeneous. In some studies, remote workers show a more sustainable idea of work demands (e.g., lower perceived time pressure; [54]), a higher quality of work relationships, and opportunities for professional development [45]. In a study, employees listed aspects of work usually referred to as positive (i.e., autonomy, individual decision making, productivity) as constitutional dimensions of e-working [28]. At the same time, telework was shown to create adverse effects on beliefs about work behaviors and roles, organization, and relationships. Telework was associated with higher job demands [34,37] and role ambiguity [37]; lower identification with the organization because of the use of ICT tools to communicate [23]; reduced effectiveness of communication among colleagues [26] and a higher conflict within the organization [55].

### 3.2. Teleworking Influences Employee Health Conditions and Work-Life Balance

Several studies showed the preventive role of teleworking for employees’ health, reporting that being a remote worker decreases employee’s levels of stress [26,44,46], negative emotions [31], strain [32], depression [22], and alcohol abuse [22].

A protective role of teleworking emerged as well. Remote workers show higher positive affective well-being and higher happiness than their colleagues [31,47], above all when they are highly resilient [56]. Interestingly, Grant and colleagues [28] showed that it is not the mere teleworking arrangement, but the ability to manage the boundaries between work life and private life when teleworking that heightens the levels of perceived well-being. Work-life balance is one of the main issues related to remote working. Current research on the role of teleworking on work-life balance showed mixed results. For example, studies focusing on positive outcomes showed a lower work-to-home conflict during teleworking days [46], even thanks to the lower time pressure due to the remote arrangement [26,54]. On the other hand, studies focusing on adverse outcomes showed an inverse relationship between remote working conditions and work-life balance, reporting higher conflict between the two [30,35,47], which was even confirmed in longitudinal studies (e.g., [34]).

At the same time, some studies highlighted risks for mental health occurring during teleworking, showing a direct link with stress, fatigue, and burnout symptoms (e.g., [48,55]). Furthermore, when support from colleagues is lacking, this connection is even stronger [39]. Within this category, Fonner and colleagues [23] described a specific type of stress occurring from interruptions during the working time due to ICT use.

Studies about physical health are heterogeneous as well. Giménez-Nadal and colleagues [44] show that being a teleworker is a protective factor for perceived physical health, as it decreases levels of pain and fatigue. However, Heiden and colleagues [55] demonstrated that an increase in the amount of time in telework is linked to higher levels of fatigue.

## 4. Discussion

The findings show that remote working conditions influence employees’ quality of work and the pleasantness of the work experience in several ways. More specifically, teleworking shapes employees’ perceptions about themselves and their workplace and contributes to their physical and mental health, particularly with regards to work-life balance.

A transversal theme that emerges from the findings is the impact of the frequency or intensity of telework during a regular work week. Although it could be hypothesized that the higher the amount of teleworking during the week, the stronger the effects on personal and work life (positive or negative), the selected papers suggest a more complex picture. Some papers underlined the positive effects of part-time teleworking, showing that employees may benefit from office-based and remote working, thus improving their professional skills and strengthening their relational bonds at work [45]. At the same time, Suh [37] reported differential risks according to the intensity of teleworking. According to the authors, low-intensity teleworkers tend to experience higher work overload, while high-intensity teleworkers tend to experience higher role ambiguity than colleagues.

Furthermore, the authors showed that low-intensity teleworkers might experience more severe difficulties and perceive their job more negatively than high-intensity telework colleagues. A possible reason for this difference, in contrast with works from Davidescu, is that part-time teleworkers may suffer from the demands occurring from both on-site work and telework. As reported, the intensity of teleworking impacts transversally all the dimensions that emerged in the results.

This heterogeneous tendency mirrors the general findings, that turn out to be mixed and varied. Such variability may be due to the specific organizational contexts in which teleworking occurs and to the individual differences of the employees involved. However, few studies addressed the role of such dimensions when studying the effects of remote work arrangements. Among these, Grant [28] showed that it is not the mere remote work arrangement but the ability to manage the boundaries between work-life and private life when teleworking that heightens the perceived well-being levels. Similarly, Bentley [32] reported that the organizational support provided to teleworkers reduces the strain perceived by employees more significantly than the telework itself. These few studies show that teleworking may generate different outcomes according to the strategic choices made within the organization when planning its introduction.

Such choices will depend, for example, on the type of organization implementing the new arrangements. Organizations will likely differ in the amount of autonomy and resources they can invest in promoting a strategic implementation of teleworking, depending on whether they are public or private, profit or non-profit, small, medium, or big. These dimensions will impact even middle managers’ roles and influence employees [57]. The same considerations can be made on the organizational sector. Studies published before the pandemic suggest that the most successful organizations implementing teleworking are mostly knowledge or sales-based or from the ICT sector [58,59], thus underlining that not all organizations can easily switch to remote working arrangements. At the same time, the pandemic forced many organizations to do so.

In a study on more than 400 organizations, Pèrez and colleagues [58] identified three main types of resources the organizations needed to pursue teleworking successfully. These are: human resources, intended as types of roles, skills, and autonomy; technological resources, such as tools, software, and equipment; organizational resources, intended as HR flexibility, management by results, and activity outsourcing. While the pandemic likely forced the acquisition of technological resources, human and organizational resources needed to be rearranged according to labor changes. Indeed, such resources can impact the implementation of teleworking, for example, by providing dedicated training opportunities for employees.

The amount of resources that organizations are willing to invest in teleworking conditions and effects will largely depend on their culture, which influences both strategical and human resources (above all regarding work-life balance, flexibility, transparency, ways and criteria for acknowledging each employee’s contribution). It is known, indeed, that teleworking arrangements have higher chances of being successful when they fit the current organizational value systems [59]. In this regard, knowledge creation and sharing are crucial to promoting a successful telework condition. The higher the consensus around the tools, dynamics, and processes embedded in remote working, the higher the chances for the employees to feel supported and experience less role conflict when they are not in the office. A particular dimension in this regard is employee autonomy and flexibility. Workers usually involved in job design and programming likely have a deep knowledge of their roles and tasks. Thanks to a higher understanding of their position and a better work organization and planning skills, the teleworking conditions may benefit their quality and experience of work in general [58].

Overall, despite being underrepresented in the current literature, the role of organizational strategies and cultures is highly promising for promoting employee health and well-being in teleworking conditions. While the pandemic created the urgency to switch to home working, organizations can now recalibrate how they manage remote working conditions. Such strategies may regard employee training, the use of tangible and intangible resources, and values.

This work may be helpful for organizations and researchers, as it provides practical and research-related suggestions.

First, findings showed that employees with different dispositions may respond differently to remote working conditions. Clearly, organizations cannot directly intervene in employees’ personal dispositions, such as stress sensitivity regarding employees’ individual conditions or skills. However, they can provide training opportunities to strengthen skills considered crucial to the personal management of teleworking arrangements, such as work-life integration and boundaries management. Interestingly, in one of the papers selected for this review, these skills emerged as constituting elements of teleworking [28]. Managers and organizational counselors may provide the organizations with training projects aimed at strengthening their employees’ soft skills. Consistently, research could provide a valuable contribution by individuating which skills are more helpful when preparing employees for teleworking, for example by elaborating skill profiles of employees showing a high degree of adaptation to this new arrangement, or by using longitudinal studies, that would allow for monitoring long-term effects of training opportunities.

Second, it is crucial for organizations to define a competitive but sustainable way to implement teleworking. For example, they could analyze how the organizational objectives can be pursued remotely, which job tasks can be completed out of the office and whether and how the employees are technically ready to do so. In other words, the “new normal” of teleworking may involve the effort to individuate the types of organizations better suit the use of teleworking and, within them, which employees (according to their job role, personal, and professional skills) may benefit themselves and the organization for using such an arrangement. Among the dimensions to be analyzed, if adequately addressed, organizational values can catalyze the effects of strategical choices, by boosting employees’ sense of belonging and meaningfulness related to the task. Even in this case, research could provide a valuable contribution, for example by individuating which organizational dimensions inform remote working arrangements.

Despite such suggestions, our work is not without limitations. Firstly, it was not possible to include COVID-related papers in the review, because the unprecedented conditions in which organizations operated during the peak of the pandemic were not comparable with the experiences before COVID, or within organizations that used this type of work arrangement even before the pandemic. Thus, we did not have the opportunity to include most studies published in the last two years, because of their strong focus on emergency-related workplace conditions. Secondly, our review did not include grey literature (e.g., organizational reports), thus potentially losing valuable content, although not published in peer review scientific journals.

## 5. Conclusions

This review considered remote working published studies previously to the COVID pandemic. The paper’s purpose was to summarize the original assumptions related to remote working, as well as the opportunities and challenges associated with its practice. We have highlighted how even before the pandemic, remote working shaped the employees’ perceptions of themselves and of the workplace, of their health, and regards their work-life balance. However, our results showed a heterogeneous picture linked to remote working’s effect on employees’ well-being and productivity. This heterogeneity can be affected by the employees’ characteristics and the organizational environments’ characteristics in which it was implemented.

We believe that our results can be double useful. First, future studies could analyze how our analysis categories evolved following COVID. In other words, how employee perceptions have changed following the pandemic.

Finally, based on remote working related benefits and risks, organizations will be able to rethink remote working in a more conscious and calibrated perspective, considering the remote working specificities, and the individual and organizational factors that are essential for its sustainable and strategic use.

## Figures and Tables

**Table 1:** Characteristics of included studies.

Authors	Paper Characteristic		Participant Characteristics		Remote Working Definition	Effects Reported		
	Year	Study Methodology	Organization (Type)	Participants		Antecedents	Positive Outcomes	Negative Outcomes
Fonner et al., 2010 [26]	2010	Quantitative	*ns*	N = 192 (89 teleworkers and 103 office-based employees)	*Telework* is a “work arrangement in which employees perform their regular work ata site other than the ordinary workplace, supported by technological connections” [27].	high-intensity telework	lower work-life conflict; higher job satisfaction	
Fonner et al., 2012 [23].	2012	Quantitative	*ns*	N = 192 (89 teleworkers and 103 office-based employees)	*Telework* is a “work arrangement in which employees perform their regular work ata site other than the ordinary workplace, supported by technological connections” [27].	high connectivity due to telework		lower identification; higher stress from interruptions
Grant et al., 2013 [28]	2013	Qualitative	five organizations across three sectors (private, public and voluntary)	N = 11 (e-workers)	*E-work* is defined as “any form of substitution of information technologies (such as telecommunications and computers) for work-related travel: moving work to the workers instead of moving workers to the work” [29]	Over-working and lack of time for recuperation		adverse impacts on well-being
Higgins et al.., 2014 [30]	2014	Quantitative	*ns*	N = 16,145 (7102 men and 9043 women)	*Telework* is “the practice of working from home, making use of the Internet, email, and the telephone” [30]	telework and high work demands		higher levels of work-to-family conflict (WFC)
Anderson et al., 2015 [31]	2015	Quantitative	US federal agency	N = 702 (teleworkers)	*ns*	teleworking days	high positive job-related affective well-being (PAWB); low negative job-related affective well-being (NAWB)	
Henke et al., 2016 [22]	2016	Quantitative	*ns*	*ns*	*Telecommuting* is “working from a home office or, less commonly, from another offsite location of the employee’s choosing” [22].	low-intensity telecommuters	significantly lower probability of depression	
Bentley et al., 2016 [32]	2016	Quantitative	28 organisations	N = 804 (teleworkers)	*Telework* is “… a flexible work arrangement whereby workers work in locations, remote from their central offices or production facilities, with no personal contact with co-workers, but the ability to communicate with co-workers usingICT” [33].	Social support and teleworker support	higher job satisfaction; lower psychological strain	
Felsted et al., 2017 [34]	2017	Quantitative	*ns*	N = 100.457	*ns*	telework	higher job satisfaction and organizational commitment	lower work-life balance (WLB)
Leung et al., 2017 [35]	2017	Qualitative	*ns*	N = 509 (teleworkers)	*Telecommuting* is a “flexible work arrangement that allows employees, usually with the aid of ICTs, to perform their tasks in various locations, primarily at home” [19,36].	low flexibility, high permeability and ICT use at home		higher family-to-work conflict (FWC)
Suh 2017 [37]	2017	Quantitative	2 global IT companies	N = 258 (teleworkers)	*Telework* is “an alternative work arrangement in which employees perform tasks elsewhere that are normally done in a primary or central workplace, for at least some portion of their work schedule, using electronic media to interact with others inside and outside the organization” [38].	technology and job characteristics		higher technostress; lower job satisfaction
Vander Elst et al., 2017 [39]	2017	Quantitative	IT company	N = 878 (employees)	*Telecommuting* is ‘‘a work practice thatinvolves members of an organization substituting a portion of theirtypical work hours to work away from a central workplace—typically principally from home—using technology to interact withothers as needed to conduct work tasks.’’ [21].	social support, participation and task autonomy	higher work-related well-being	
Giovanis 2018 [40]	2018	Quantitative	*ns*	*ns*	*Flexible working arrangements* are “flexible work schedules are interventions which enable greater control to the employees, providing psychological and tangible resources to enhance well-being” [41,42,43]	Flexible employment arrangements	higher workplace performance	
Giménez-Nadal et al., 2019 [44]	2019	Quantitative	*ns*	N = 43,374 (22,083 males; 21,291 females)	*ns*	male teleworkers	lower levels of pain, tiredness and stress	
Davidescu et al., 2020 [45]	2020	Mixed methods	*ns*	N = 220 (employees)	*ns*	partial home working	higher organizational performance, social and professional relationships, learning, work motivation	
Delanoeije et al., 2020 [46]	2020	Quantitative	Engineering	N = 78 (39 intervention group; 39 control group)	*Home-based telework* is “a work arrangement that allows employees to execute working tasks from home during some portion of the working week using information and communication technologies” [19].	teleworking days	lower stress and work to-home conflict; higher engagement and performance	
Kazekami 2020 [47]	2020	Quantitative	*ns*	N = 9200 (regular employees)	*ns*	Teleworking commuters or low-intensity teleworkers	higher productivity; higher life satisfaction	
Song et al., 2020 [48]	2020	Quantitative	*ns*	*ns*	*Telework* corresponds to “conducting formal, paid work at home during normal business hours, a majority of homeworkers are not typical teleworkers and do not have a formal agreement with their employers” [26,49,50,51,52,53].	telework		lower subjective well-being; higher stress level
Darouei et al., 2021 [54]	2021	Quantitative	Legal, academia, IT	N = 34 (professional workers)	*Telecommuting* is “a policy that enables employees to perform their job at home during some part of the week and stay connected to the office by means of communication technologies” [21].	Teleworking day	lower time pressure and work-family conflict	
Heiden et al., 2021 [55]	2021	Quantitative	Public universities	N = 392 (academics)	*ns*	high-intensity telework		higher stress and conflict within the organization
Kapoor et al., 2021 [56]	2021	Quantitative	*ns*	N = 326 (employees)	*Telework* is “the work that can be operated from any location of employees’ convenience from where they can perform their duties using technology and applications” [56].	perceived stress, telework (mediator)		higher psychological stress, lower psychological well-being

Note. *ns* = not specified. In “Telework definition” is reported the definition referred to by the authors in each paper.
